# Preparation and Characterization of Submicrometer and Nanometer Cellulose Fiber with Biogenic SiO_2_

**DOI:** 10.3390/polym17060761

**Published:** 2025-03-13

**Authors:** Yakoub Touati, Dora Kroisová, Rawaa Yahya, Štěpánka Dvořáčková

**Affiliations:** Assembly and Engineering Metrology, Department of Machining, Faculty of Mechanical Engineering, Technical University of Liberec, 461 17 Liberec, Czech Republic; dora.kroisova@tul.cz (D.K.); rawaa.yahya@tul.cz (R.Y.); stepanka.dvorackova@tul.cz (Š.D.)

**Keywords:** nanocellulose, renewable resources, rice husk, silica, wet grinding, thermal stability, chemical properties

## Abstract

This study aims to explore the feasibility of producing submicrometer and nanometer cellulose fibers derived from rice husk treated with a novel method which selectively eliminate hemicellulose and lignin, while maintaining the integrity of the cellulosic and silica constituents. Three distinct processing methods are tested to extract the nanocellulose, namely hand milling, ball milling, and wet milling using a high-shear wet media mill from Masuko Sangyo Co., Ltd., Kawaguchi-city, Japan. A range of analytical methods, including Scanning Electron Microscopy (SEM), Energy-Dispersive X-ray Spectroscopy (EDX), Transmission Electron Microscopy (TEM), Fourier Transform Infrared Spectroscopy (FTIR), Differential Scanning Calorimetry (DSC), and Thermogravimetric Analysis (TGA), are utilized to characterize the morphology, elemental composition, thermal stability, and chemical properties of the samples. The study revealed that among the tested methods, only wet milling successfully produced cellulose nanofibrils and silica nanoparticles, forming a biogenic organic–inorganic nanohybrid system. The nanofibers had lengths in the range of 120 nm and below, while the nanoparticles were in the tens of nanometers. The silica nanoparticles were found to adhere to the cellulose nanofibrils, forming a biogenic organic–inorganic nanohybrid system, with potential applications across diverse fields, including biomedical (drug delivery, biosensing, bone regeneration, and wound healing), cosmetic (skin and dental care), technical (insulating aerogels, flame retardants, and UV-absorbing pigments), and food applications (dietary supplements, thickeners).

## 1. Introduction

Utilizing agricultural waste and by-products for industrial applications presents a viable solution to environmental and economic challenges. One interesting example of agricultural waste is rice husk, which, according to FAO (2022), generates approximately 155 million tons of husks [1].

After the processing of harvested rice, 20–30% of the grain weight remains as rice husks [2]. These husks are composed of various components, including 25–35% cellulose, 18–21% hemicellulose, 26–31% lignin, 15–17% silica, 2–5% other inorganic elements, and about 7.5% moisture, depending on the geographical region and rice species [3].

Among these components, cellulose stands out as a renewable, biodegradable, non-toxic, and water-insoluble polysaccharide. It occurs naturally in the fibrous structures of plant cell walls, where it is synthesized through the process of photosynthesis [4].

Interestingly, cellulose is not exclusively derived from photosynthetic organisms. It can also be synthesized by non-photosynthetic cells, such as those in certain fungi and mantles [5]. In rare cases, cellulose can also be produced by some bacteria [6]. This remarkable polymer represents the most abundant renewable source of biodegradable material on Earth, with an estimated annual production of 10¹¹ to 10¹² tons via photosynthesis [7].

Plant cellulose forms filaments averaging 3 nm in diameter, composed of approximately 36 crystalline, parallel b-1-4-glucan chains [8]. The microfibrils tightly surround the cells forming the cell wall and provide structural integrity by resisting external stresses and internal osmotic pressures. Additionally, cellulose microfibrils are interconnected with other cell wall polymers, such as hemicelluloses and pectins.

The cellulose chains in plants are formed at the plasma membrane by the addition of UDP-glucose units by enzymatic structures called terminal complexes (TCs). These complexes are formed by rosettes in the case of plants. Rosettes (CelS complexes) are formed by aggregates of the polypeptide CesA [9] (The CESA complex (CER1-SBE1-SBE2-SBE3-SEC14) is a group of proteins that play a key role in cellulose biosynthesis in plant cells. This complex consists of five subunits: CER1, SBE1, SBE2, SBE3, and SEC14.

Together, these subunits catalyze the synthesis of cellulose by UDP-glucose, a precursor of cellulose, and transport it to the plasma membrane where it polymerizes to form microfibrils [9].

After its formation, the chains cluster to form cellulose microfibrils stabilized by intermolecular hydrogen bonds [9], forming type I cellulose chains with parallel glucose chains. This type of structure is metastable, meaning that it is not thermodynamically most favorable. Type II cellulose shows a different hydrogen bonding system because of the existence of an intermolecular hydrogen bond between the OH groups of C6 and C2 of another chain, the intramolecular bonding of OH in C2 is avoided and an intermolecular hydrogen bond of OH–C2 to OH–C2 of the next chain is formed, the cellulose II molecules are more densely packed and strongly interbonded and, therefore, cellulose II is less reactive [7]. Type I occurs much more frequently than type II and is divided into two types of crystal structures, Iα e Iβ, which differ in the arrangement of intermolecular hydrogen bonds. These two types of cellulose occur in different proportions in nature [9]. There are 3 other types of cellulose structure (III, IV, and V), but those are only produced by chemical processes and do not occur in nature [9].

Building on this natural abundance of cellulose, nanocellulose (NC) emerges as a promising derivative. Nanocellulose is a natural fiber obtained from cellulose, characterized by a fiber diameter usually less than 100 nm and a length reaching up to several micrometers. Nanocellulose is also a biodegradable material with a density of approximately 1.6 g/cm^3^ [10], that also exhibits a large surface area to volume ratio. As a result, nanocellulose can potentially exhibit those advantages, including improved interfacial interaction when used as a filler in composites, uniform distribution in thin films, a large surface area for catalyst or active molecule immobilization, greater potential for chemical modification or functionalization, and effective oil absorption for applications like oil spill cleanup [11].

It also exhibits excellent mechanical properties. It has a high stiffness, its modulus of elasticity reaches up to 220 GPa, which is higher than that of aramid fibers. In addition, nanocellulose has a high tensile strength of up to 10 GPa, which is higher than cast iron (100 MPa to 350 MPa) [12]. The strength-to-weight ratio is 8 times higher than that of stainless steel [12]. In addition, nanocellulose is transparent and full of reactive hydroxyl groups that can be functionalized to affect various surface properties.

Nanocellulose can be divided into:

- Cellulose nanocrystals, also known as nanocrystalline cellulose (CNC), are rigid short structures. The geometric dimensions of CNCs can vary widely in diameter from 2 nm to 20 nm and in length from 100 nm to 500 nm [12], and the degree of crystallinity in CNCs ranges from 55% to 88%. The dimensions and crystallinity of a given CNC depend on the source of cellulose and the conditions of extraction [12].
- Cellulose nanofibrils (CNF), also known as nano-fibrillated cellulose (NFC), have a long and flexible structure. CNFs consist of stretched bundles (aggregates) of elementary nanofibers [13], which are made up of alternating crystalline and amorphous regions. CNFs can have a diameter in the range of 20 nm–50 nm and a length in the range of 500 nm–2000 nm and the degree of crystallinity of CNFs ranges from 50% to 60%.
- HCNC is a new type from the group of nanocelluloses synthesized by van de Ven and his colleagues by oxidation of the so-called cellulose periodate (periodate-oxidized cellulose, HlO_4_). Hairy nanocelluloses consist of a crystalline rod-like body with amorphous loose chains (“hairs”) at both ends, which are more accessible than the crystalline segment for chemical reactions and which also enhance colloidal stability [13].

Because nanocellulose properties are determined by both its source and extraction process, it is essential to examine these methods after considering its various origins.

These methods fall into two primary categories: Top-Down and Bottom-Up [14]. While the Top-Down approach starts with the production of nanocellulose from bulk cellulose, the Bottom-Up approach starts with the production of nanocellulose from dissolved cellulose or monomers [14].

A common example of a Top-Down approach is the three-step purification process used for isolating nanocellulose from plant sources [15]. The first step is purification of the raw material to remove non-cellulosic components from the plant material and isolation of the purified cellulose. Purification can be carried out, for example, with sodium or potassium hydroxide followed by bleaching with sodium chlorite [15]. This procedure is usually repeated several times to obtain the purest cellulose possible but leads to the destruction of the silica contained in the rice husk.

The second step is a controlled chemical treatment, usually hydrolysis with acid or oxidation with TEMPO (2,2,6,6-tetramethyl piperidine-1-oxides). This treatment helps to cleave amorphous regions, remove local interfibrillar crystalline bonds and facilitate the extraction of nanocellulose [15]. After these two steps, the third step is the isolation of the nanocellulose itself. This is conducted, for example, by dissolution in organic solvents or mechanically mainly by high-pressure homogenization, microfluidization, or grinding [4].

This research focuses on synthesizing and characterizing a functional, naturally derived nanocomposite of cellulose nanofibrils and biogenic silica nanoparticles. Unlike typical nanocomposites, our material integrates both organic and inorganic components in a ready-to-use form. Utilizing silica from rice husk provides an abundant, sustainable source and ensures biocompatibility, broadening applications, particularly in biomedical and food sectors. One particularly interesting potential use is in dental materials, especially when the nanostructures are interconnected, such as those formed by cellulose nanofibrils and silica nanoparticles, which can enhance the mechanical properties while being biocompatible [16].

## 2. Materials and Methods

**Figure 1:**
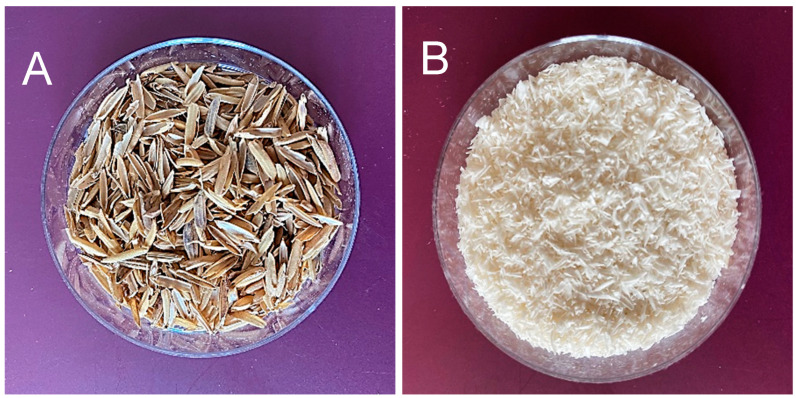
Raw rice husks (**A**), rice husk after treatment (**B**).

For this experiment, an organic–inorganic material system of natural origin was provided by Cersum s.r.o. (Liberec, Czech Republic)—Figure 1. The material used is obtained via a novel treatment of rice husk and is characterized by the simultaneous presence of cellulose fibers and nanoparticles of biogenic silica—Figure 1.

Following treatment, the rice husks were rinsed with distilled water until reaching a neutral pH. The resulting material was subsequently employed as input for hand milling and other grinding techniques.

**Figure 2:**
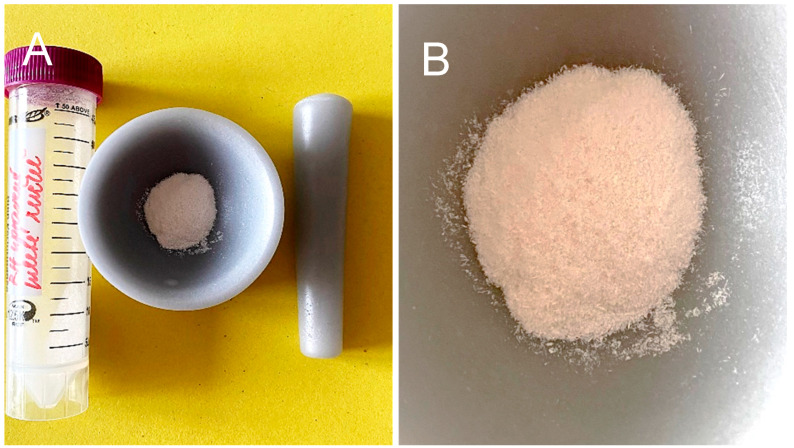
Corundum bowl with pestle (**A**) and output material after hand grinding (**B**).

Three distinct processing methods are used to obtain nanocellulose, namely hand milling in a corundum friction bowl with a hammer for approx. A total of 15 min at normal laboratory temperature and humidity—Figure 2.

**Figure 3:**
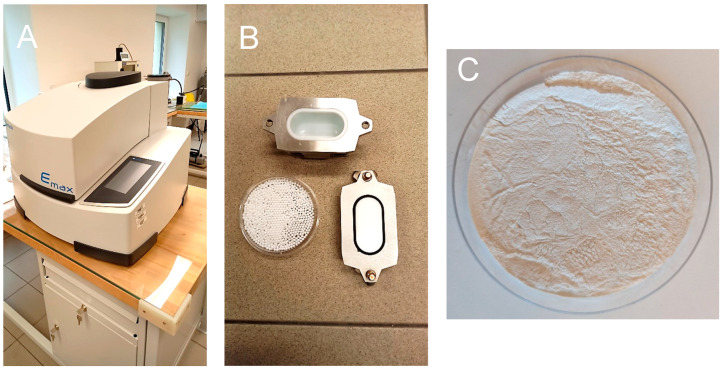
Emax ball mill with components—grinding bowl and grinding balls (**A**,**B**), output material after grinding by ball mill (**C**).

A ball mill manufactured by Retch and labeled Emax was used. It is a mill suitable for grinding a wide range of materials (alloys, ceramics, glass, metal oxides, wood, cellulose), as it combines high-frequency impact, intense friction and controlled circular movements of the vessel in which the grinding balls are placed together with the sample—Figure 3.

The input material in this experiment was dry treated rice husk. Milling was carried out in a ceramic cup with 3 mm diameter ceramic balls, both made of zirconia. The grinding of the sample was carried out at a speed of 1500 rpm for 15 min. The resulting material was a fine white powder—Figure 3.

**Figure 4:**
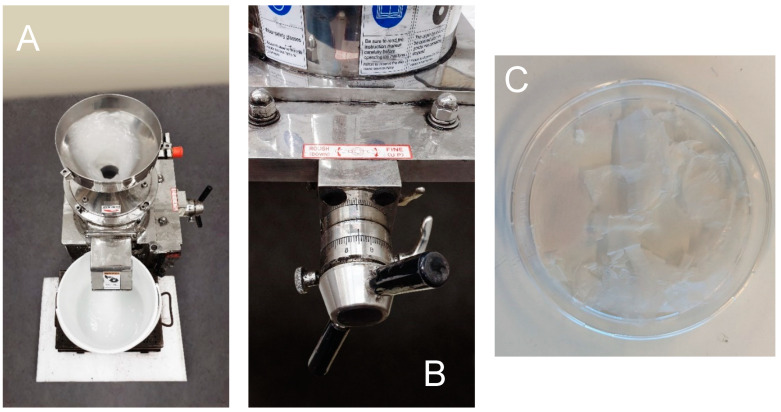
Ultra-fine friction grinder “Supermasscolloider” from Masuko Sangyo Co., Ltd., Japan (**A**) control mandrel (**B**), output material after wet-grinding and drying (**C**).

Lastly used the “Supermasscolloider” device from Masuko Sangyo Co., Ltd., Japan—Figure 4. This machine uses two superimposed ceramic discs, of which the upper disc is static, and the lower disc is dynamic, rotating at a speed of 1996 rpm. The distance between the discs was gradually reduced by a hand-operated mandrel to the maximum achievable value, which was −100 µm. The raw material (treated rice husks) was mixed in distilled water, which served as a cooling and lubricating medium to prevent its degradation due to high temperatures caused by frictional forces and, at the same time, to obtain a product in suspension. The final product was then dried—Figure 4.

Among the three grinding methods employed, wet milling is anticipated to yield the smallest fiber dimensions due to the higher shear stress it generates, effectively facilitating defibrillation. Table 1 lists the used samples for the characterization.

### 2.1. Characterization

#### 2.1.1. SEM and EDX

The nature and structure of all samples were examined using scanning electron microscopes (FE SEM Zeiss ULTRA Plus, Carl Zeiss Microscopy GmbH, Jena, Germany and TESCAN MIRA3, TESCAN GROUP a.s., Brno, Czech Republic). As all samples were non-conductive, they were coated with a 4 nm layer of Pt-Pd (Quorum Q150R ES, Quorum Technologies Ltd., Lewes, UK) prior to microscopic examination. A Zeiss Stemi DV4 stereomicroscope (Carl Zeiss Microscopy GmbH, Jena, Germany) was used for sample preview and preparation.

As for the basic chemical composition or elemental analysis, it was carried out using energy dispersive X-ray analysis (EDX). The analysis was carried out as part of the microscopic evaluation of the samples.

#### 2.1.2. TEM

Transmission electron microscope (TEM) was used to evaluate the nanostructures obtained after wet milling of the samples. The samples were analyzed in the Advanced Materials Analytics Laboratory, Palacký University in Olomouc

#### 2.1.3. FTIR

Fourier transform infrared spectroscopy was recorded using a Nicolet iS10 infrared spectrometer (Thermo Fisher Scientific, Fisher Scientific, Waltham, MA, USA) in the range of 400–4000 cm^−1^. The samples were dried and then manually ground to a fine powder in a corundum friction bowl with a hammer.

#### 2.1.4. Thermal Analysis

The thermogravimetric analyses were performed using a Mettler Toledo TGA2 (Mettler-Toledo (Switzerland) GmbH, Greifensee, Switzerland) in a temperature range from 0 to 600 °C in a nitrogen atmosphere, then after 600 °C the inert atmosphere was lifted. The heating rate was set at 10 °C/min throughout the process.

DSC analyses were performed using a Mettler Toledo DSC 1/700 (Mettler Toledo, Switzerland) over a temperature range of 0 °C to 500 °C, and the test was conducted in a nitrogen atmosphere with a heating rate of 10 °C/min.

The weight of the samples used was determined using a Mettler Toledo XSE 105 Dual Range analytical balance (Mettler-Toledo (Switzerland) GmbH, Greifensee, Switzerland).

## 3. Results and Discussion

### 3.1. SEM and EDX

- Raw rice

**Figure 5:**
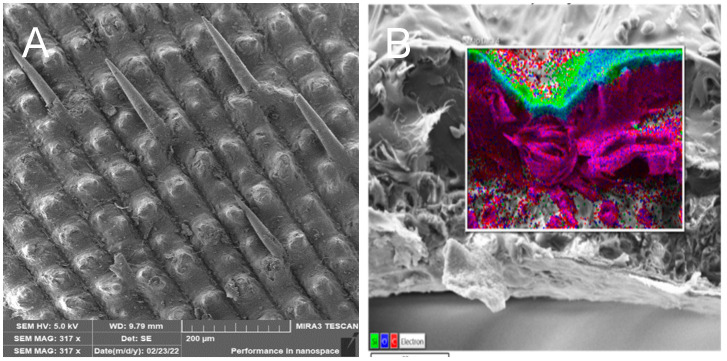
Surface morphology of raw rice husk (**A**); transverse splitting of the husk showing the line of high silica content on the surface (**B**) EDX [3].

The inner part of the husk is made up of parallel fibers forming a compact surface that is in contact with the rice grain—Figure 5.

**Figure 6:**
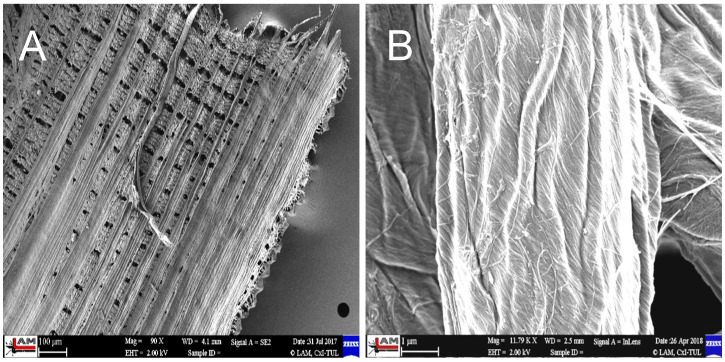
Inner part of rice husk after treatment (**A**), close-up of the surface of a single nanofiber after treatment (**B**) [3].

**Figure 7:**
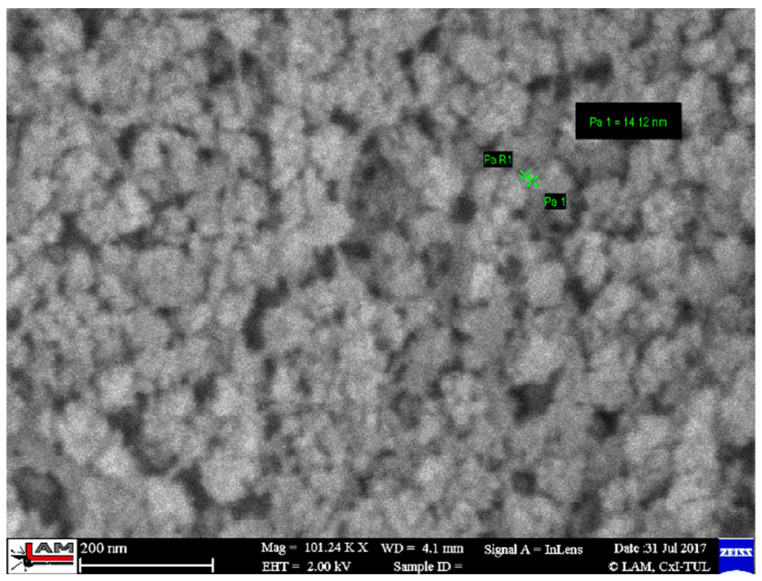
Morphology of cellulose nanofibrils with silica nanoparticles after rice husk treatment [3].

**Figure 8:**
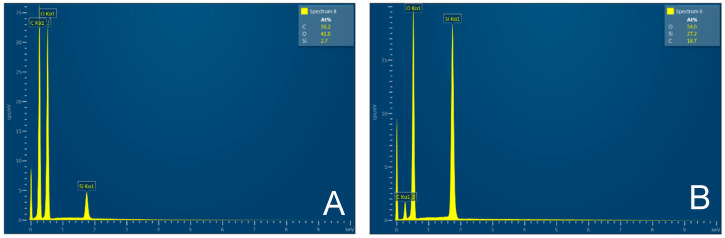
EDX analysis of cellulose fibers (**A**); nanoparticles after rice husk treatment (**B**). EDX [3].

The SiO_2_ particles that complement the fibers are very small, ranging in size from 10 to 20 nm—Figure 6. The individual particles form spherical clusters with dimensions in the order of tens of nanometers—Figure 7. The presence of an organic phase or nanofibers among the silica nanoparticles is evident from EDX analysis—Figure 8.

From the above analyses, it is evident that both microfibers/nanofibers and silica nanoparticles can be obtained from the treated husks in a single process, which are directly interconnected with the fibers to form an organic–inorganic complex system—Figure 5 and Figure 6. The importance of the obtained materials lies in the biogenic nature of the silica nanoparticles, which are very well absorbed by the human organism, for which the presence of silicon as a trace element is essential.

- RHS—Untreated rice husk

**Figure 9:**
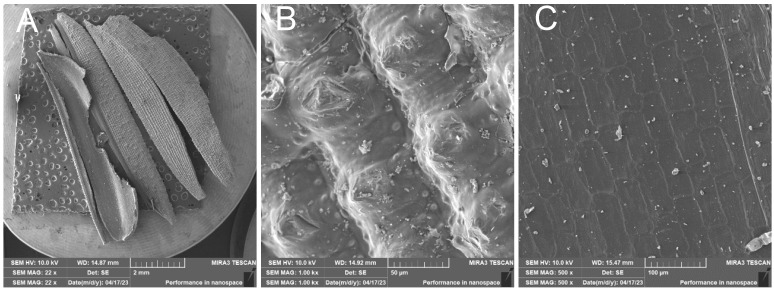
Raw rice husk—overview image (**A**); close-up of the compact outer surface of the rice husk formed by the silica crust (**B**); close-up of the inner surface of the husk (**C**).

**Figure 10:**
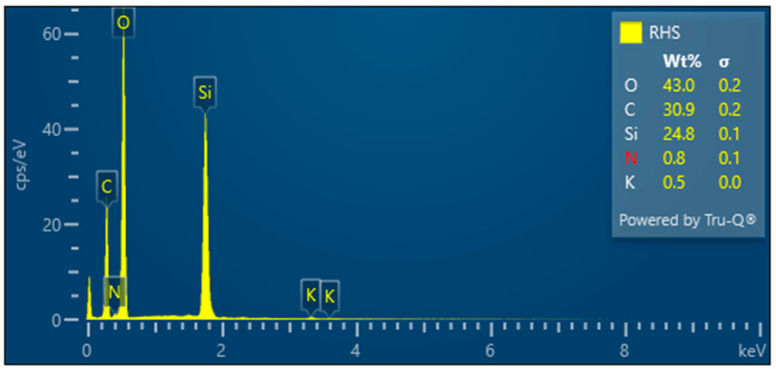
Rice husks raw—EDX analysis indicates the presence of oxygen, carbon, high amounts of silicon, and small amounts of potassium and sodium.

The surface of the rice husk is covered with a compact hard inorganic crust made up of silica nanoparticles. In contrast, the inner part of the husk is smooth—Figure 9. The EDX analysis—Figure 10 confirms the chemical composition of the rice husk and the presence of inorganic elements.

- RHU—Treated rice husk

**Figure 11:**
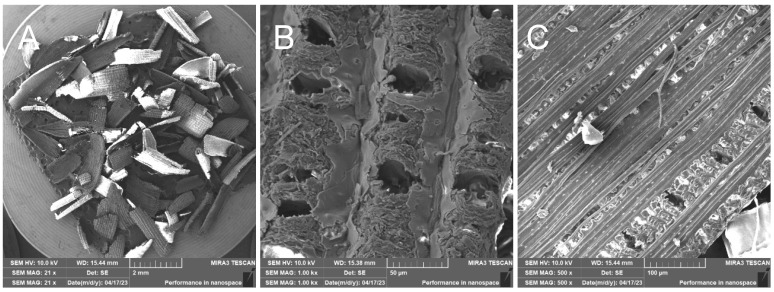
Rice husk treated—overview image (**A**); close-up of outer surface of rice husk with broken silica crust (**B**); close-up of inner surface of husk with visible fibers (**C**).

**Figure 12:**
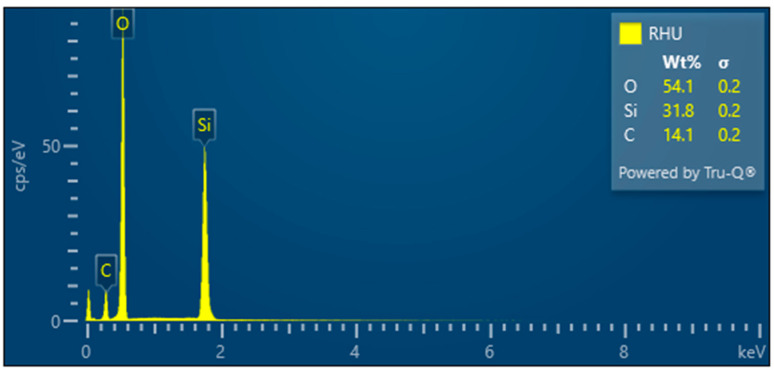
Rice husk treated—EDX analysis indicates the presence of oxygen, carbon, and high amounts of silicon. Other elements were not analyzed.

Compared to the untreated raw rice husk, the treated husk has a different character. The outer part of the husks has a disrupted inorganic surface—Figure 11, while the inner organic surface—Figure 11, is made up of parallel fibers. The treated rice husks have also a different presence ratio of elements—Figure 12—carbon, oxygen, silicon, which is due to the compositional modification following the treatment.

- RHUR—Treated rice husk hand milled

**Figure 13:**
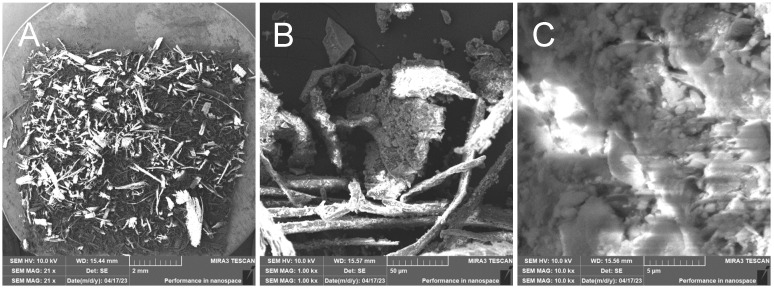
Hand-milled rice husks—overview image (**A**); close-up of the sample with visible fibers and larger formations caused by breakage of the outer inorganic crust (**B**); close-up of the broken inorganic crust (**C**).

**Figure 14:**
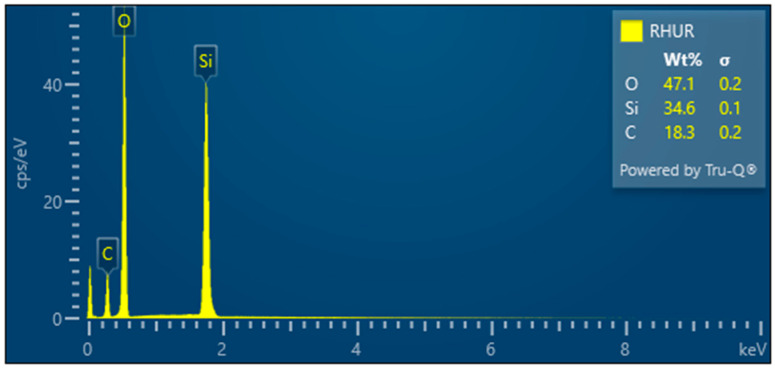
Rice husks hand milled—EDX analysis indicates the presence of oxygen, carbon, and high amounts of silicon.

The treated rice husks were subjected to different types of milling. The simplest method is manual milling—using a friction bowl and pestle. The fibers are partially crushed, and the surface siliceous layer is also crushed—Figure 13. EDX analysis shows a high proportion of silica in the sample—Figure 14.

- RHUM—Treated rice husk ball mill milled

**Figure 15:**
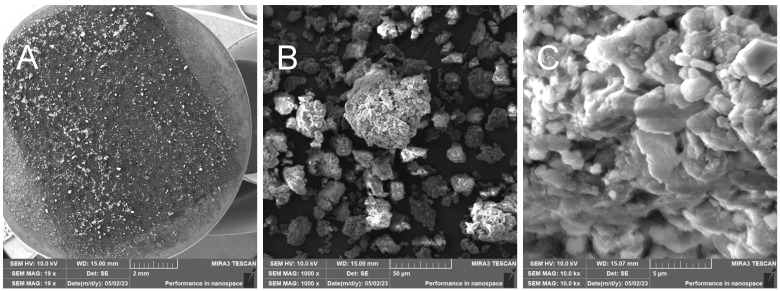
Rice husks ground in a mill—overview image of the prepared sample (**A**); close-up of the resulting powder without fibers (**B**); close-up of the powdered material (**C**).

**Figure 16:**
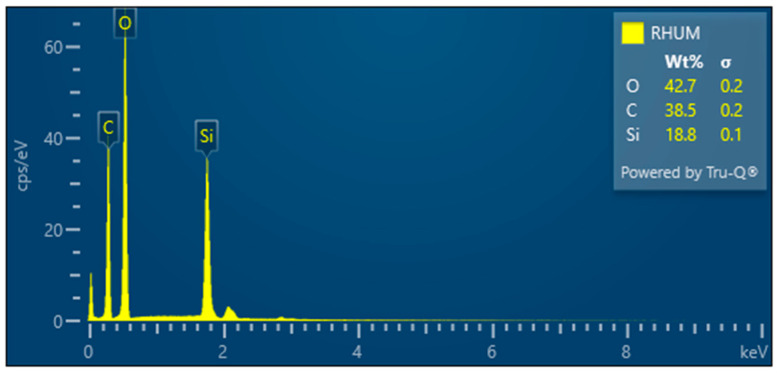
Rice husks ground in a mill—EDX analysis indicates the presence of oxygen, carbon, and high amounts of silicon.

The grinding of treated rice husks in a ball mill results in the crushing of the fibers and inorganic crust into small particles—Figure 15. This method is not suitable for obtaining the fibrous structure. EDX analysis documents the presence of elements in the sample—Figure 16.

- RH1C—Treated rice husk wet ground, 1 cycle

**Figure 17:**
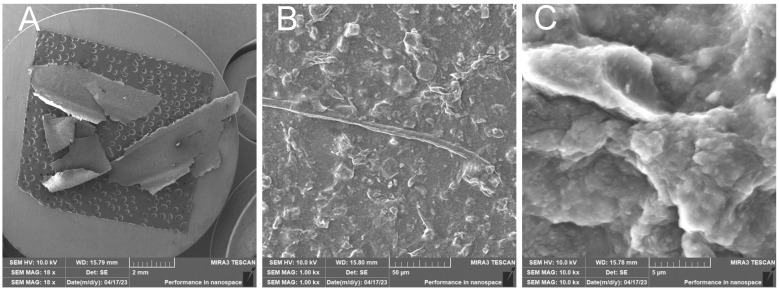
Rice husk wet-milled in a nanomill after 1 cycle—overview image of the dried sample (**A**); close-up image of the surface of the prepared sample with undestructed cellulose fiber and surface formed by broken inorganic crust (**B**); close-up image (**C**).

**Figure 18:**
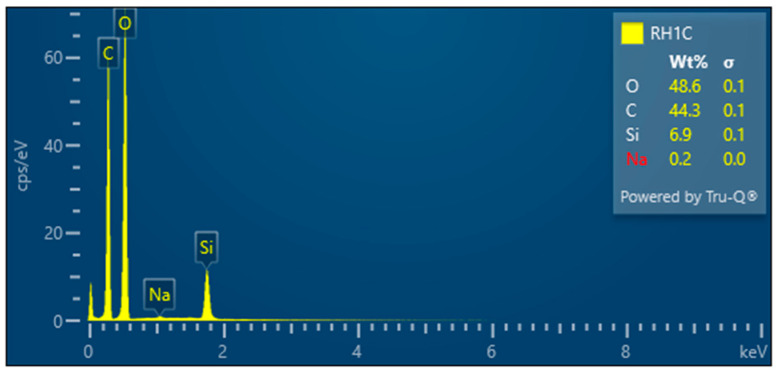
Rice husk wet-milled in a nano-mill after 1 cycle—EDX analysis indicates the presence of oxygen, carbon, silicon, and a small amount of sodium.

After 1 cycle, the wet-grinded rice husk yields a sample characterized by the presence of roughly crushed inorganic crust—Figure 17, showing the unfibrillated cellulose fiber. EDX analysis provides information on the chemical composition—Figure 18.

- RH15C—Treated rice husk wet ground, 15 cycles

**Figure 19:**
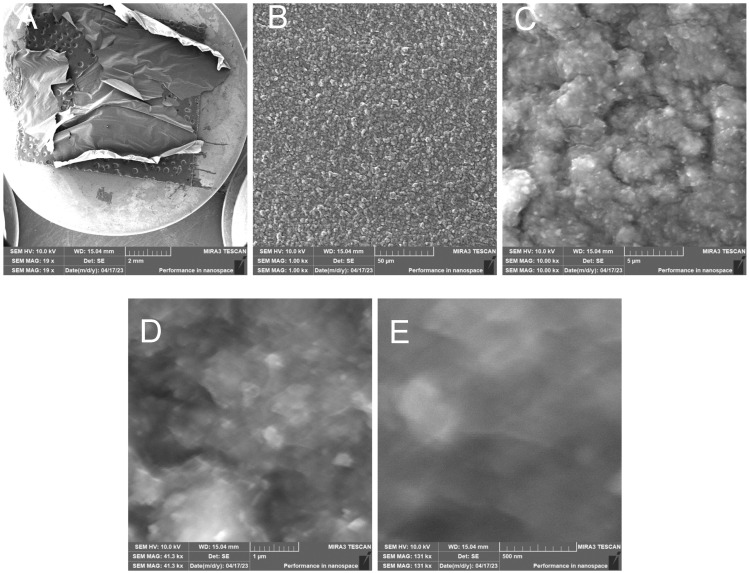
Wet-milled rice husks after 15 cycles—overview image of dried sample (**A**); overview image of sample surface (**B**); close-up image of sample surface (**C**); close-up image of surface with visible submicrometer particles (**D**); close-up (**E**).

**Figure 20:**
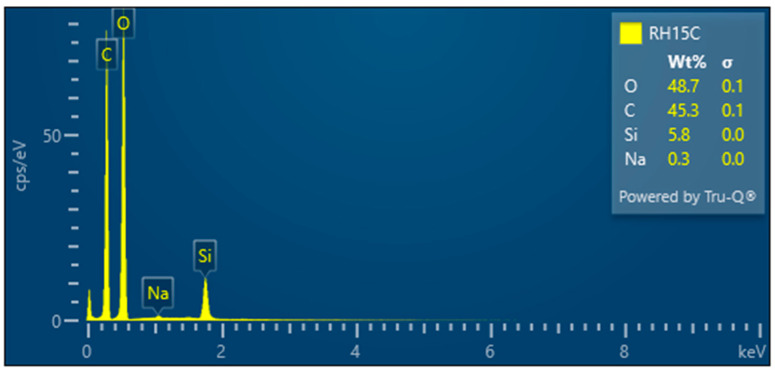
Rice husk wet-milled in a nano-mill after 15 cycles—EDX analysis indicates the presence of oxygen, carbon, silicon, and a small amount of sodium.

The microscopic images of the samples after 15 cycles of wet grinding in the nano-grinder show the highest crushing degree of the surface inorganic crust, the particles are very fine, and the images show submicrometer/nanometer silica particles as well as the presence of very fine, nanometer-scale filaments connecting these particles—Figure 19. EDX analysis provides information on the chemical composition—Figure 20.

Microscopic images obtained from wet milled samples of treated rice husk from the nano-grinder show that organic—cellulose fibers are interconnected with silica nanoparticles. The amount of silica varies, see Table 2.

EDX analysis revealed significant information on the silicon (Si) content of the raw and modified rice husk (RH) samples. Initially, the raw RH had a Si mass fraction of approximately 25%, which is consistent with previous studies and the literature [17,18].

However, after removing the lignocellulosic part of the RH, the Si content increased to approximately 32%, which means an increase in the Si mass ratio of 28%. This increase was also observed for the hand-milled RHUR sample.

Subsequently, the RHUM sample was subjected to ball milling and the Si content was approximately 18.8%, corresponding to approximately 60% of the original Si mass ratio. However, after wet milling, the Si mass ratio dropped significantly to 7%, which represents approximately 21% of the original Si content. The observed low SiO_2_ content in the nanocellulose samples after the wet milling process can be attributed to two main factors. the separation of cellulose and SiO_2_ particles due to the high shear force applied in the mill and the strong tendency of nano SiO_2_ to agglomerate [19]. These factors lead to agglomeration and sedimentation of SiO_2_ particles in the final suspension, which explains the low SiO_2_ content and the problem of achieving homogeneous dispersion in the studied samples subjected to the wet milling process.

The observed higher Si content in RH10C is attributed to the potential agglomeration of Si particles. In contrast, the 16% reduction in Si content in RH15C, relative to RH1C and RH5C, indicates a potential inhomogeneous distribution of Si within the sample. This variability poses a challenge for applications requiring a uniform and high Si concentration when used as a filler to ensure the expected appropriate mechanical properties. Although sampling errors are a possibility, subsequent analyses will aim to validate these observations and explore solutions to ensure consistent Si distribution.

### 3.2. TEM

**Figure 21:**
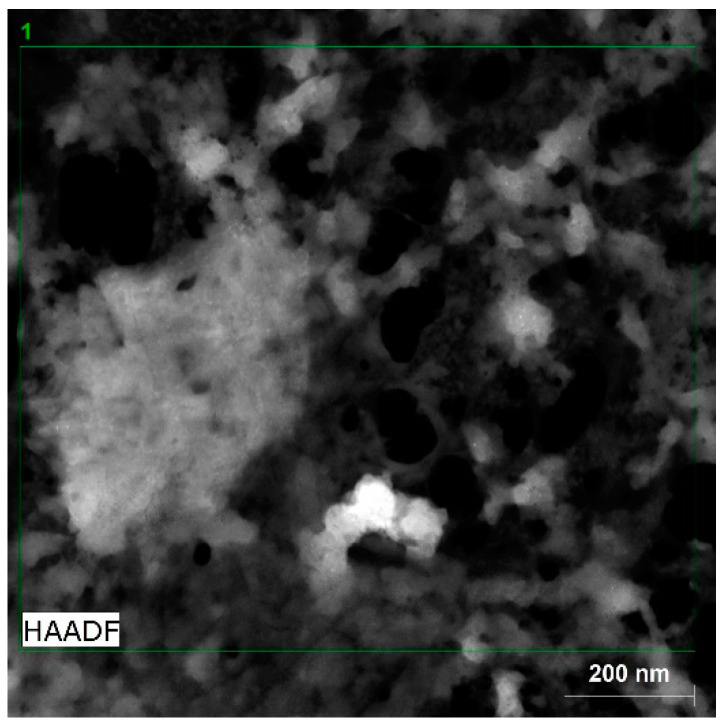
TEM mapping.

The TEM mapping image—Figure 21—shows the distribution of the elements of the RH15C sample into three-dimensional formations in the sample. The bright regions correspond to the SiO_2_ cluster regions.

**Figure 22:**
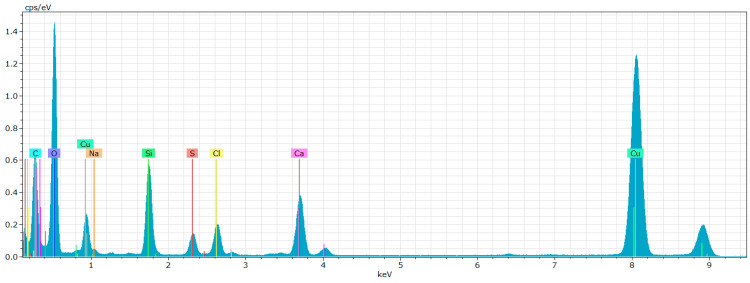
Wet-milled rice husks after 15 cycles—EDX analysis indicates the presence of oxygen, carbon, silicon, and other contaminating elements.

The EDX analysis presented in Figure 22 demonstrates the presence of carbon, oxygen, and silicon within the samples. This convergence of results from both SEM and TEM-EDX strengthens the validity of our elemental identification. Notably, the EDX data confirms an elevated presence of silica, supporting our prior hypothesis regarding nano-SiO_2_ agglomeration.

**Figure 23:**
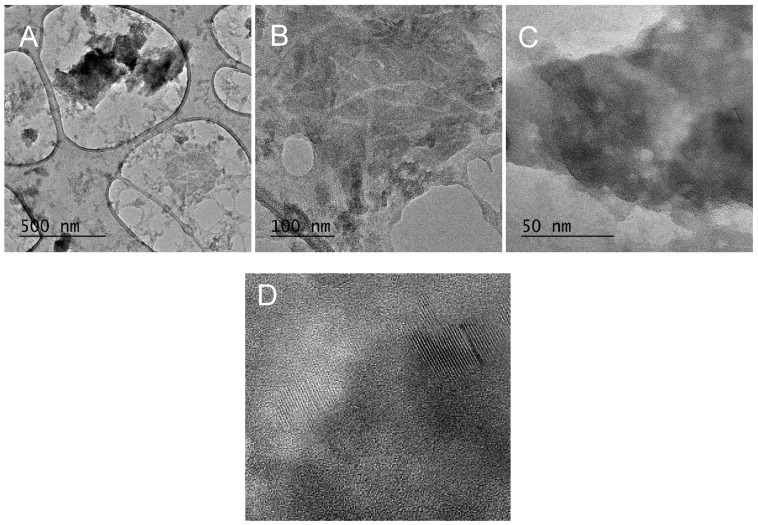
Wet-milled rice husks after 15 cycles—TEM image of nanofibers and SiO_2_ cluster (**A**), cellulose nanofibrils (**B**), ordered structures (**C**), close-up of ordered structures (**D**).

This observation is visually reinforced by the TEM image in Figure 23, which further supports the existence of cellulose nanofibrils with an approximate 10 nm fiber diameter.

Furthermore, the observed presence of ordered structures in the sample—Figure 23—could potentially be attributed to silica polymorphs such as: quartz, cristobalite, and tridymite.

However, the detection of calcium, sodium and chlorine in the EDX—Figure 22—indicates possible contamination of the sample.

### 3.3. FTIR

**Figure 24:**
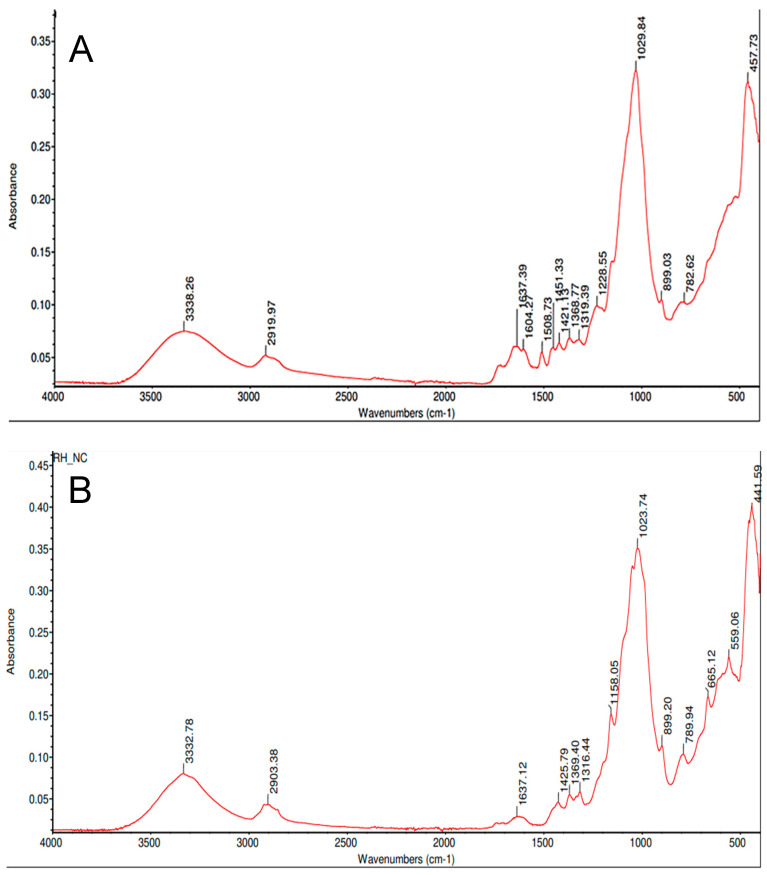
FTIR spectrum of natural rice husk (**A**); FTIR spectrum of cellulose post-treatment and wet-milling (**B**).

The broad absorption band in Figure 24 with a maximum at 3338 cm^−1^ can be attributed to O-H valence vibrations in water molecules with hydrogen bonds or to OH groups present in cellulose, hemicellulose, and lignin [20]. The absorption band at 2919 cm^−1^ is attributed to asymmetric and symmetric valence vibrations in the C-H bonds in the –CH3 and –CH2 groups of lignin, cellulose, and hemicellulose structures [20]. The band at 1637 cm^−1^ is due to both the deformation vibrations of the water molecules (δ-H_3_O) and the presence of C=C bonds in the organic components [20]. The band at 1508 cm^−1^ is characteristic of the vibration of C=C bonds in the aromatic rings of lignin [20]. The absorption bands at 1368, 1319, and 1228 cm^−1^ for rice husk may be related to the OH groups of lignin and polysaccharides that form the husk structure [20]. The intense band with a maximum at 1029 cm^−1^ corresponds to the valence vibrations of silicon oxygen (SiO_4_) tetrahedra [21]. The peak at 899 cm^−1^ corresponds to β-glycosidic bond [22]. The absorption band at 782 cm^−1^ is assigned to symmetric stretching vibrations of SiO_4_ tetrahedra and the band at 457 cm^−1^ corresponds to bending vibrations of Si-O [23].

The absorption values at 3333, 2903, 1637, 1369, 1158, and 1023 cm^−1^ in the spectrogram in Figure 24 are associated with native cellulose. The broad band with a maximum at 3333 cm^−1^ is attributed to stretching of hydroxyl groups [24]. The absorption band at 2903 cm^−1^ is due to C-H stretching [25]. The band at 1637 cm^−1^ is due to H-O-H bending of absorbed water [25]. The peak at 1369 cm^−1^ corresponds to O-H bending and the peak at 1158 cm^−1^ is attributed to the C-O antisymmetric bridge stretching [25]. The non-cellulosic polysaccharides were almost completely eliminated as shown by the absence of the peak at 1 210 cm^−1^ [25]. In addition, a weak band at 665 cm^−1^ can be discerned, which is according to Didik et al. [23] characteristic of crystalline cristobalite, which forms at temperatures around 1400 °C [26]. Nonetheless, to perform an unambiguous identification of either cristobalite or tridymite microcrystals, it is required the combination of local probe techniques that could be correlated with the corresponding high-quality images. Possibly, the most robust and widely used technique for phase identification is powder XRD [27]. FTIR absorption values are identified in Table 3. 

### 3.4. DSC

**Figure 25:**
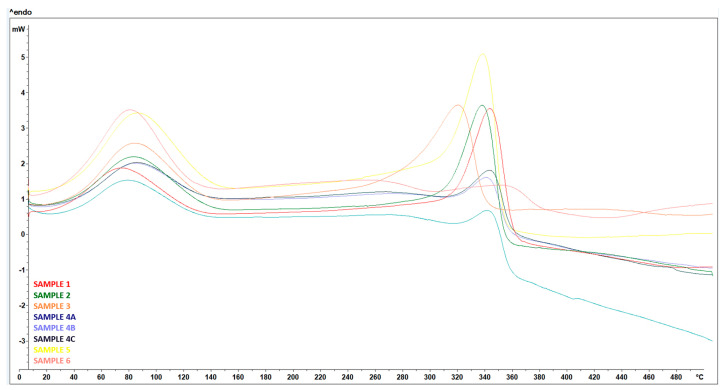
DSC curves—Treated RH (SAMPLE 1, SAMPLE 2), treated RH ball milled (SAMPLE 3) samples after wet grinding after 15 cycles (SAMPLE 4A, 4B, 4C), treated RH hand-milled (SAMPLE 5), and untreated RH (SAMPLE 6).

During the DSC analysis, all samples showed similar behavior below 100 °C, with an endothermic peak observed in the temperature range 60–90 °C, corresponding to the evaporation of moisture contained in the samples—Figure 25, which was also reflected in the subsequent TGA.

Subsequently, the untreated RH sample was characterized by two exothermic peaks, the first at 296 °C and the second at 411 °C, which match the temperature range of hemicellulose (160–360 °C) and lignin (180–900 °C) decomposition, respectively [28].

The treated RH samples, including hand-milled and ball-milled, showed one large endothermic peak at a temperature range of 313 °C to 338 °C, respectively. This peak corresponded to cellulose degradation occurring in the temperature range of 240–390 °C [28]. Unlike hemicellulose and lignin, cellulose degradation is an endothermic event.

Wet-milled samples showed a small exothermic event at a temperature ranging from 304 °C to 308 °C, which could be attributed to residual hemicellulose, followed by an endothermic event at about 307 °C corresponding to cellulose degradation. In contrast to the other samples, a significant inverse trend was observed; the negative DSC values indicate an exothermic characteristic associated with cellulose pyrolysis reactions. From 337 to 500 °C, the DSC curve decreased significantly with further temperature increase. This can be attributed to the cleavage of some functional groups in the cellulose residue [29].

### 3.5. TGA

From the thermogravimetric curves, additional information to previous analyses (SEM, EDX, and DSC) can be obtained in order to refine the values of the amount and ratio of organic and inorganic phases in the sample.

**Figure 26:**
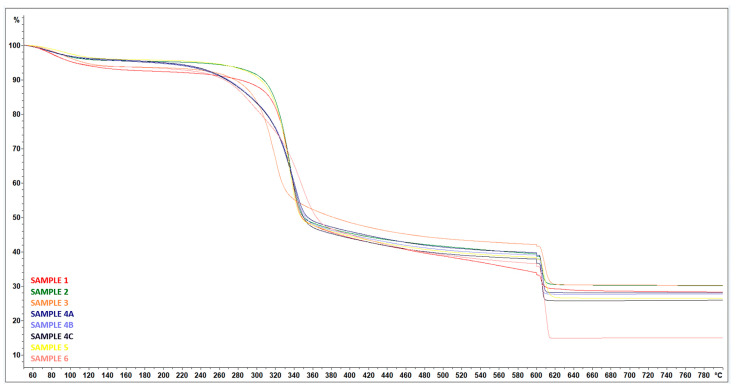
TGA curves—Treated RH (SAMPLE 1, SAMPLE 2), treated RH ball milled (SAMPLE 3) samples after wet grinding after 15 cycles (SAMPLE 4A, 4B, 4C), treated RH hand-milled (SAMPLE 5), and untreated RH (SAMPLE 6).

The mass loss of all samples was observed in four different phases—Figure 26. The first phase, which took place between room temperature and approximately 120 °C, was due to evaporation of the moisture contained in the samples. This loss was found to represent approximately 4% to 7% of the total weight loss.

In the second phase, a sharp decrease in weight of approximately 43% to 50% was observed. This significant weight loss was due to the thermal decomposition of hemicellulose and cellulose [30].

The third phase was characterized by a gradual weight loss of 6% to 11%. This weight loss can be attributed to the decomposition of lignin, which occurs over a wide temperature range [28].

The last phase, which took place between 600 °C and 800 °C, was attributed to the disruption of the inert atmosphere, with a decrease of between 5% and 22%—Table 4.

The main distinguishing factor in the case of untreated and treated samples is observed in the final residue from the TG analysis. All the treated RH samples have a final residue higher than 25%, while the untreated material has a final residue below 15%—Table 4. The lower residue of the untreated material can be attributed to its thermal instability, which is evident from the DSC analysis, which could be due to impurities in the material, or an increase in carbon production due to the increased number of free end chains in the treated samples. The end chains begin to decompose at lower temperatures, increasing the amount of carbon produced [31].

Another differentiating factor between the samples was the steeper gradient of the nanocellulose curves (Samples 4A, 4B, 4C), which can be attributed to the lower thermostability compared to the other samples, as confirmed by the lower onset degradation temperature T_0.5_—Table 4. The unique morphology of the nanocellulose samples, which is characterized by high SSA (specific surface area), may contribute to the lower thermostability. Indeed, the increased surface area may allow a greater reactivity and promote faster degradation. In addition, it has been reported that nanocellulose acts as an efficient pathway for phonons, leading to its higher thermal conductivity [32]. The better thermal conductivity of nanocellulose can be attributed to the less scattering of phonons in the bundle of crystallized cellulose chains in nanocellulose [33].

## 4. Conclusions

In conclusion, wet milling with a “Supermasscolloider” emerged as the sole effective method for cellulose nanofibrils production from a readily available agricultural waste source. The resulting nanofibers exhibited targeted dimensions (hundreds of nanometers in length, tens of nanometers in diameter) as verified by SEM and TEM analyses. However, attaining these nanoscale dimensions appears to come at a cost to thermostability, likely due to the distinctive morphology of the nanofibers, which exhibit a high specific surface area and consequent enhanced thermal conductivity. Nevertheless, the resulting material represents a unique biogenic organic–inorganic particle-fiber system, where cellulose fibers remained bonded to biogenic silica nanoparticles. Intriguingly, TEM and FT-IR analyses detected the presence of crystalline silica polymorphs, indicating localized temperature spikes potentially reaching up to 1400 °C during grinding. These findings suggest that greater control over the grinding process can be achieved. This includes minimizing silica dispersion and optimizing the process for improved energy efficiency and yield. However, the characterization methods employed in this study lack the precision needed to definitively identify the specific polymorph present. This novel method for producing cellulose nanofibrils alongside silica nanoparticles presents significant potential for research advancement in various fields. Further exploration and refinement are warranted to fully exploit its potential and address any associated challenges.

## Figures and Tables

**Table 1:** Abbreviations and definitions of samples.

Abbreviation	Definition	Sample
RHU	Treated rice husks	SAMPLE 1&2
RHUM	Treated, ball mill milled	SAMPLE 3
RH1C	Treated, wet ground 1 cycle	-
RH5C	Treated, wet ground 5 cycles	-
RH10C	Treated, wet ground 10 cycles	-
RH15C	Treated, wet ground 15 cycles	SAMPLE 4A & 4B & 4C
RHUR	Treated, hand-milled	SAMPLE 5
RHS 1	Untreated rice husks	SAMPLE 6

**Table 2:** Si content in samples.

Sample	Weight Proportion Si [%]
RHS	24.8
RHU	31.8
RHUR	34.6
RHUM	18.8
RH1C	6.9
RH5C	6.9
RH10C	16.6
RH15C	5.8

**Table 3:** Identification of different FTIR absorption values.

Absorption Value	Identification
3333	Stretching of hydroxyl groups
2903	C-H stretching
1637	H-O-H bending of absorbed water
1508	Vibration of C=C bonds in the aromatic rings of lignin
1369	O-H bending
1158	C-O antisymmetric bridge stretching
665	Crystalline cristobalite

**Table 4:** TGA onset degradation temperature and final residue.

Samples	Degradation Onset Temperature T_0.5_ [°C]	Residue [%]
Untreated	273.49	14.81
Treated	300.00	26.38
SAMPLE 1	303.24	28.46
SAMPLE 2	305.27	30.15
SAMPLE 3	283.79	30.22
SAMPLE 4A	263.75	27.96
SAMPLE 4B	264.34	27.56
SAMPLE 4C	263.86	25.67

## Data Availability

The original contributions presented in this study are included in the article. Further inquiries can be directed to the corresponding author.
